# Effects of fatigue are more pronounced on the uninjured side during single‐limb jumps 2 years after an acute Achilles tendon rupture

**DOI:** 10.1002/ksa.70579

**Published:** 2026-08-21

**Authors:** Unnur Sædís Jónsdóttir, Roy Tranberg, Karin Grävare Silbernagel, Annelie Brorsson, Kristín Briem

**Affiliations:** ^1^ Department of Orthopaedics, Institute of Clinical Sciences at Sahlgrenska Academy University of Gothenburg Gothenburg Sweden; ^2^ Department of Physical Therapy University of Delaware Newark Delaware USA; ^3^ Department of Physical Therapy, Research Centre of Rehabilitation and Movement Science, School of Health Sciences University of Iceland Reykjavik Iceland

**Keywords:** Achilles injury, biomechanics, countermovement jump, exertion, joint power

## Abstract

**Purpose:**

Achilles tendon rupture (ATR) is a serious lower limb injury that may result in persistent deficits in strength and endurance of the calf muscles. Compensatory mechanisms at proximal joints are known, but the effects of fatigue are less studied. The aim was therefore to evaluate the effect of a fatigue protocol on lower limb kinetics during performance of single‐limb drop countermovement jumps (CMJ) in individuals with a history of a unilateral ATR.

**Methods:**

Thirty participants were recruited on average 2 years after ATR. Drop CMJs were performed on each side before and after a fatigue protocol, and peak power values for the hip, knee and ankle joints were calculated, as well as jump height and duration. A mixed model analysis of variance was used to evaluate inter‐limb differences pre‐ and post‐fatigue during the landing (eccentric) and push‐off (concentric) phases of the involved (INV) and uninvolved (UNinv) side.

**Results:**

The fatigue protocol had a greater effect on peak ankle joint power of the UNinv side across both phases (by a mean [95% confidence interval, CI] of 1.06 [0.58–1.54] W/kg) compared to the INV side (0.51 [0.03–1.00] W/kg; *p* = 0.039). Moreover, greater fatigue‐induced changes in ankle joint power were found during landing (by a mean [95% CI] of 1.13 [0.64–1.61] W/kg) compared to push‐off (0.45 [0.04–0.93] W/kg; *p* = 0.011), across both sides. A trend towards increased knee joint power was seen as a response to fatigue across both sides and phases (*p* = 0.062), with no change at the hip.

**Conclusions:**

Rehabilitation following ATR should emphasize progressive reliance on the INV side across the rehabilitation continuum by incorporating strength, endurance and training under fatigued conditions. This approach may help reduce compensatory reliance on the UNinv side, potentially lowering the risk of overuse‑related injuries.

**Level of Evidence:**

Level III.

**Abbreviations**
ATR: Achilles tendon rupture
ATRS: Achilles tendon rupture score
BMI: body mass index
CMJ: countermovement jump
INV: involved side
LSI: limb symmetry index
PAS: Physical Activity Scale
UNinv: uninvolved side

## INTRODUCTION

Acute Achilles tendon rupture (ATR) is a common injury with increasing incidence [12, 14, 19]. The typical individual who sustains an ATR is a male injured while playing a sport that requires rapid change of directions [16, 18]. As most of those who rupture their Achilles tendon are active individuals, it is unfortunate that only about 70%–80% return to play after the injury [2, 35]. Reasons for not returning can vary but are likely, in part, due to reduced performance on the injured side [1, 7, 8, 28] and lack of full functional recovery [21].

Studies assessing lower limb biomechanics after ATR have shown a compensatory shift, where power is transferred away from the ankle to the knee and hip [20, 28], and this has been found to persist even as long as 6 years after injury [8]. Long‐term deficits in calf muscle functional performance are well known and Achilles tendon elongation [11, 17] as well as muscle atrophy of the triceps surae [4, 23, 38] are likely sources of impaired function, although proximal compensatory strategies may overcome those deficits to maintain performance of sport‐related tasks such as jumping [34].

The effects of fatigue on lower limb performance after an ATR are not well documented in this population compared to those with anterior cruciate ligament injuries, where mental and physical fatigue have gained attention [5]. Due to the persistent impairments known to affect individuals after ATR, it is important to identify the effects of fatigue on lower limb function and joint loading. In healthy populations, studies have found that fatigue may induce a variety of changes in lower limb biomechanics during single‐leg landing tasks, with many showing reduced vertical ground reaction forces and decreased hip and knee joint moments [30]. In individuals with a history of ATR, recognizing what effects fatigue may have on lower limb joint loading and performance is clinically relevant, and the knowledge may inform rehabilitation during the return‐to‐sports continuum.

Therefore, the primary aim of the study was to determine the effects of fatigue on lower limb kinetics in terms of peak joint power of the ankle, knee and hip, during a single‐leg drop countermovement jump (CMJ) performed 1–3 years after an ATR. Fatigue was hypothesized to result in disproportionate lowering of peak power at the ankle joint on the injured side compared to the contralateral side, with a concomitant shift towards increased peak knee and hip joint power. Secondary outcomes, used to reflect the influence of fatigue on jump performance, were jump height and time‐to‐peak ankle push‐off power.

## MATERIALS AND METHODS

### Participants

For this study, a total of 30 participants with midportion rupture of the Achilles tendon were recruited from local physiotherapy clinics. Participants were between 18 and 65 years old; 24 (80%) were male and were treated with surgery (*n* = 12 [40%]) or non‐surgically (*n* = 18 [60%]; Table 1). The injury had to have occurred 1–3 years prior to the evaluations, as participants were required to perform demanding functional tasks. Treatment had to be initiated within 4 days of injury, as delayed diagnosis influences patient management decisions. The study focused on primary ATR and limb symmetry during functional performance, and so volunteers were excluded if they had re‐ruptured the Achilles tendon or a history of ATR on the contralateral side, or if they had physical impairments that could affect the functional assessment.

**Table 1:** Demographics of the whole group (*n* = 30).

Variables	Mean (SD)	Median	Range
Age, years	43 (8.9)	44	24–59
Time since rupture, months	24.3 (8.3)	27	12–35
Height, cm	176 (6.5)	177	164–187
Weight, kg	80.7 (9.8)	81.6	59.8–96.8
BMI, kg/m^2^	26.0 (2.4)	26.4	21.4–30.6

*Note*: Values are presented as means and standard deviation (SD).

Abbreviation: BMI, body mass index.

Prior to enrolment and data collection, information about the research was provided, and participants gave oral and written consent. The research protocol was approved by the Regional Ethical Review Board before initiation of the study (nr 803‐15).

### Patient‐reported outcome measurements

The Achilles tendon total rupture score (ATRS), a 10‐item questionnaire, was used to assess symptoms and functional recovery. The final score is on a scale of 0–100, with the score of 100 being equivalent to full function and no symptoms [25]. An ordinal scale from 1 to 6 was used to measure physical activity, with a score of 6 being heavy physical activity at least three times a week, while a score of 1 signifies no physical activity (modified Physical Activity Scale [PAS]) [13, 29].

### Physical evaluation

Participants' tendon length was measured bilaterally using standard ultrasound images [32], after which their leg length was measured as the distance from the crista iliaca to the floor.

A total of 54 reflective markers were attached to the participants. Anatomical markers were put on proximal and distal ends of the defined segments: feet, shanks, thighs, pelvis and the trunk, while marker clusters were used to track each segment. A single force plate (Kistler AG) and a 12‐camera motion capture system (Oqus 4; Qualisys AB) were used for data acquisition, sampling at 1000 and 250 Hz, respectively. An initial static measurement was done, followed by the removal of 16 static markers prior to the warmup.

The participants completed a 5‐min warm‐up on a treadmill prior to the functional evaluation, completing two minutes of level‐ground walking at a self‐selected brisk walking speed and another three minutes with a 5% incline.

To evaluate jump performance after ATR, the single‐leg drop CMJ was used [20, 28, 34]. The protocol set up for the present study was that the participants stood on one leg on a 20 cm high box, dropped down to the force plate that was embedded in the floor, landed on the same leg and then jumped directly up in the air again (Figure 1). The instructions were to place both hands on their hips and jump as high as they could immediately after landing on the force plate. Participants were allowed a few test jumps for familiarization before the actual testing took place, first on the uninvolved (UNinv) side, followed by the involved (INV) side. Verbal instructions were standardized for all the participants, as well as the footwear that they used (Omega; Bagheera). Following the pre‐fatigue drop CMJ, the heel‐rise test was conducted to measure performance, while also serving to induce local fatigue. For both the drop CMJ and heel‐rise tests, the UNinv side was tested first, followed by the INV side.

**Figure 1:**
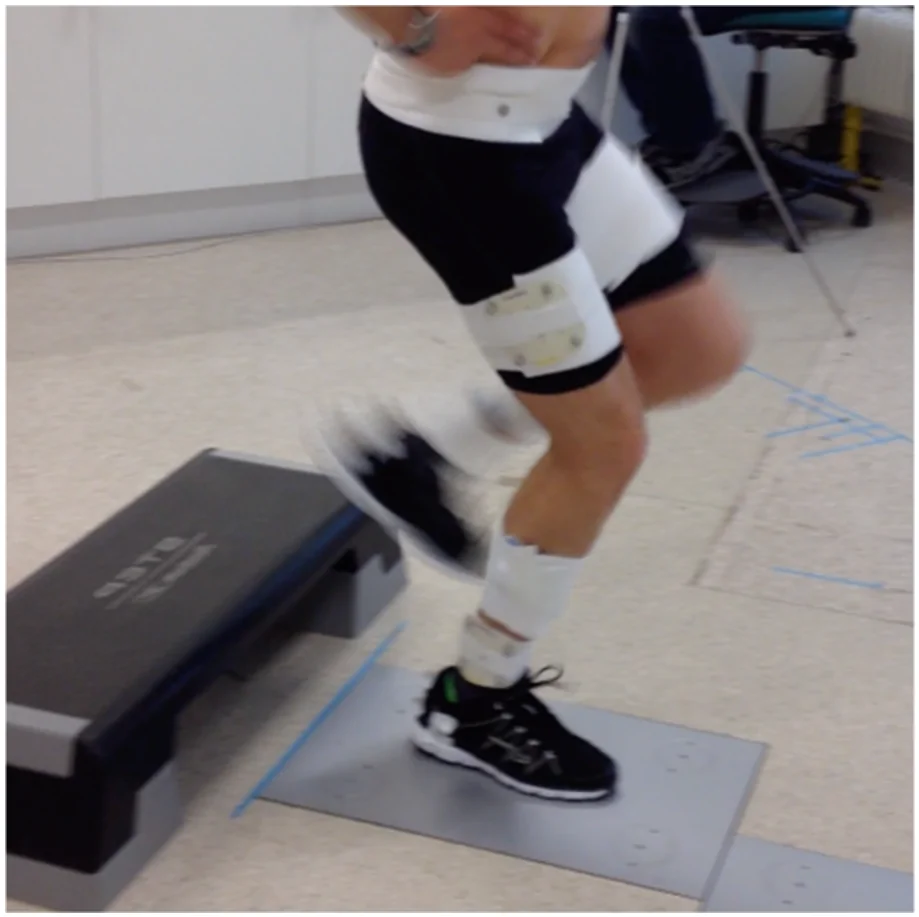
Single‐leg drop countermovement jump (drop off a 20 cm high box, immediately jumping maximally up).

The heel‐rise test measured work (body weight × distance covered = joules), height (cm) and number of repetitions. The test is valid and widely used to evaluate performance after ATR and for those with Achilles tendinopathy [31]. A linear encoder from MuscleLab (Ergotest Technology) was used for data collection, where the UNinv side was tested first, followed by the INV side. Participants stood on one leg on a 10° incline on top of a box with the linear encoder attached to the heel of the shoe. They were allowed to touch the wall with their fingertips for balance, and a metronome was used to maintain a frequency of 30 heel‐rises per minute. The instructions were to lift as high as possible and lower the heel down to level between lifts. The aim was to do as many heel‐lifts as possible, but the test was stopped if the participant could not lift more than 2 cm, or could not maintain the set frequency, or stopped.

A 5‐min lower limb exertion protocol on a slide board was used to induce lower limb fatigue [6]. Participants had to slide from side to side by pushing off boards that were individually adjusted according to their leg length (multiplied by 1.5). Before initiating the protocol, they were familiarized with the Borg ratings of perceived exertion, a 15‐point scale ranging from 6 to 20 [9]. The standardized instructions were to maintain their hips and knees flexed while pushing and sliding from side to side, bringing their feet together at each edge of the slide board. At the end of every minute, the participant was instructed to go a little faster and was told how many minutes were left. During the last minute, the participant was instructed to maximize their pace. The goal was that all the participants would reach between 15 and 20 points, corresponding to hard to maximal exertion on the Borg scale, by the end of the 5 min. After completion, the drop CMJs were repeated in the same fixed order as previously.

### Data processing and analysis

Commercial biomechanical software (Visual 3D™; C‐Motion, Inc) was used to process the data. Ground reaction force data were filtered with a low‐pass Butterworth filter with a 20 Hz cut‐off to filter noise from the data. A low‐pass Butterworth filter with an 8 Hz cut‐off was then used for the marker data. The trials were labelled accordingly using fatigue (pre‐ and post‐fatigue) and sides (INV and UNinv). Joint power was calculated for bilateral ankles, knees and hips in the sagittal plane as well as in the frontal plane for the hip, and absolute peak values within the eccentric (landing) and concentric (push‐off) phases were extracted for analysis. The presented kinetic data were normalized to body weight. Jump height was measured based on the highest point of the pelvis segment after push‐off; jump duration was defined as the time from landing until the peak push‐off power; and phases were identified according to the lowest position of the pelvis during the stance phase on the force plate.

Limb symmetry indices (LSIs) were calculated for heel‐rise test performance parameters, and Achilles tendon length (INV/UN × 100) and descriptive statistics were presented as mean (standard deviation [SD]) or median (interquartile range [IQR]). Statistical analysis was performed using Jamovi (Version 2.7.22), using a mixed model to assess effects of the fatigue protocol (pre vs. post) on joint power of each lower limb (INV vs. UNinv) during each phase of the drop CMJ (landing vs. push‐off). Achilles tendon length and limb symmetry were used as covariates in statistical models. For statistical power of 95% and alpha = 0.05, 25 participants were needed for the landing phase (eccentric power), and 28 participants were needed for the push‐off phase (concentric power) to detect side‐to‐side difference at the ankle [8].

## RESULTS

### Self‐reported and functional outcome measures

The median (IQR) physical activity of 4 (1) indicated moderate exercise for 1–2 h per week or easier physical activities for 4–5 h per week. The ATRS ranged from 56 to 100 points with a median (IQR) of 88 (11). As expected, tendon elongation was apparent on the INV side, but heel‐rise test performance varied greatly (Table 2). The median number of reps was similar between limbs, but when factoring in heel‐rise height for each rep, the inter‐limb differences in the amount of work were much clearer (Table 2). Self‐reported fatigue reached the target of 15 on the Borg scale for all participants by the end of the 5‐min protocol (median [IQR] 17 (1)). Biomechanical data were only available for 27 of the initial cohort due to technical issues discovered during data processing (marker drop‐out, shifting of the rigid clusters used for tracking). Two others did not complete the self‐reported measures, but their functional performance measures were included in the analysis.

**Table 2:** Mean (standard deviation) or median (interquartile range) and range for heel‐rise test performance on the involved (INV) and uninvolved (UNinv) sides, and for ultrasound‐measured tendon length.

	INV side	UNinv side	LSI (%)
Heel‐rise height (cm)	11.3 (2); 7.3–16	13.5 (1.9); 9.2–17.5	83.9 (12.0); 55.7–107.6
Heel‐rise repetitions	31 (10.5); 16–49^a^	29.5 (12.5); 17–65^a^	95.8 (16.4); 57.0–129.4
Heel‐rise work (Joule)	1960.5 (836); 1287–3737^a^	2361 (1109); 1310‐6014^a^	82.8 (21.1); 29.3–122.7
Achilles tendon length (cm)	23.6 (1.9); 20.6–27.5	22.5 (2); 18.9–27.3	105 (10.2); 93.6–122^a^

Abbreviation: LSI, limb symmetry index (INV/UNinv × 100).

^a^ Median (interquartile range). Heel‐rise deficits on the INV side are reflected by LSI values < 100, and relative tendon lengthening of the INV side by LSI values > 100.

### Biomechanical assessment

Statistical main effects showed that jump height declined only marginally overall after the fatigue protocol (*p* = 0.052) and that participants generally jumped slightly higher on the UNinv side (by a mean [95% CI] of 29 [25–33] mm; *p* < 0.001). However, a fatigue‐by‐side interaction (*p* < 0.001) showed minimal pre‐ to post‐fatigue change on the INV side, while the UNinv side showed a slight drop in jump height post‐fatigue (mean [95% CI] inter‐limb difference in pre‐ to post‐change of 8 [6–12] mm). Similarly, time‐to‐peak was generally shorter on the UNinv side (*p* < 0.001) and overall lengthened in response to fatigue (*p* < 0.001). Again, a fatigue‐by‐side interaction (*p* = 0.017) showed that the fatigue protocol induced slower UNinv side performance (time to peak), exceeding measured changes on the INV side (mean [95% CI] inter‐limb difference in change of 14 [3–26] ms).

Descriptive statistics and results of analyses regarding the effects of fatigue on lower limb joint power at the ankle, knee and hip are shown in Table 3. Data for pre‐ and post‐fatigue power for the landing and push‐off phases of the single‐limb drop CMJ are shown for each joint.

**Table 3:** Mean and standard deviation (SD) for joint power (W/kg; absolute values), pre‐ and post‐fatigue protocol during landing (eccentric) and push‐off (concentric).

		Landing; mean (SD)		Push off; mean (SD)	
Joint	Fatigue	Involved	Uninvolved	Involved	Uninvolved
Ankle^a^, ^b^, ^c^, ^d^, ^e^	Pre	11.4 (4.2)	14.0 (3.0)	8.7 (2.1)	11.9 (2.4)
	Post	10.5 (3.8)	12.5 (2.7)	8.5 (2.2)	11.0 (2.2)
Knee^a^, ^c^	Pre	10.4 (3.6)	9.7 (2.6)	5.9 (2.6)	5.4 (1.9)
	Post	10.8 (3.5)	9.7 (2.7)	6.1 (2.8)	5.3 (1.8)
Hip sag^c^	Pre	4.9 (4.1)	4.6 (2.9)	3.8 (2.1)	3.6 (2.1)
	Post	5.1 (4.2)	4.8 (3.0)	3.9 (2.4)	4.0 (2.3)
Hip frontal^c^	Pre	3.7 (1.6)	4.1 (1.9)	1.9 (1.0)	1.7 (0.9)
	Post	3.7 (1.7)	3.6 (1.6)	1.8 (1.0)	1.9 (1.0)

Abbreviations: kg, kilograms; sag, sagittal; W, watts.

^a^ Side *p* < 0.001.

^b^ Fatigue *p* < 0.001.

^c^ Phase *p* < 0.01.

^d^ Fatigue × Side *p* < 0.05.

^e^ Fatigue × Phase *p* < 0.05.

Statistical main effects at the ankle show clear deficits on the INV side (*p* < 0.001), with greater eccentric (landing) than concentric (push‐off) power (*p* < 0.001), and that ankle joint power generally decreased after the fatigue protocol (*p* < 0.001) across both phases and sides. A fatigue by side interaction, however, showed that ankle joint push‐off power decreased to a greater extent on the UNinv side (by a mean [95% CI] of 1.06 [0.58–1.54] W/kg) compared to the INV side (0.51 [0.03–1.00] W/kg), across both phases (*p* = 0.039; Figure 2). The degree of relative Achilles tendon elongation did not impact this interaction (*p* = 0.341). A fatigue‐by‐phase interaction showed that lowering of ankle joint power following the fatigue protocol was greater during landing (by a mean [95% CI] of 1.13 [0.64–1.61] W/kg) compared to push‐off (by 0.45 [0.04–0.93] W/kg), across both sides (*p* = 0.011; Figure 2).

**Figure 2:**
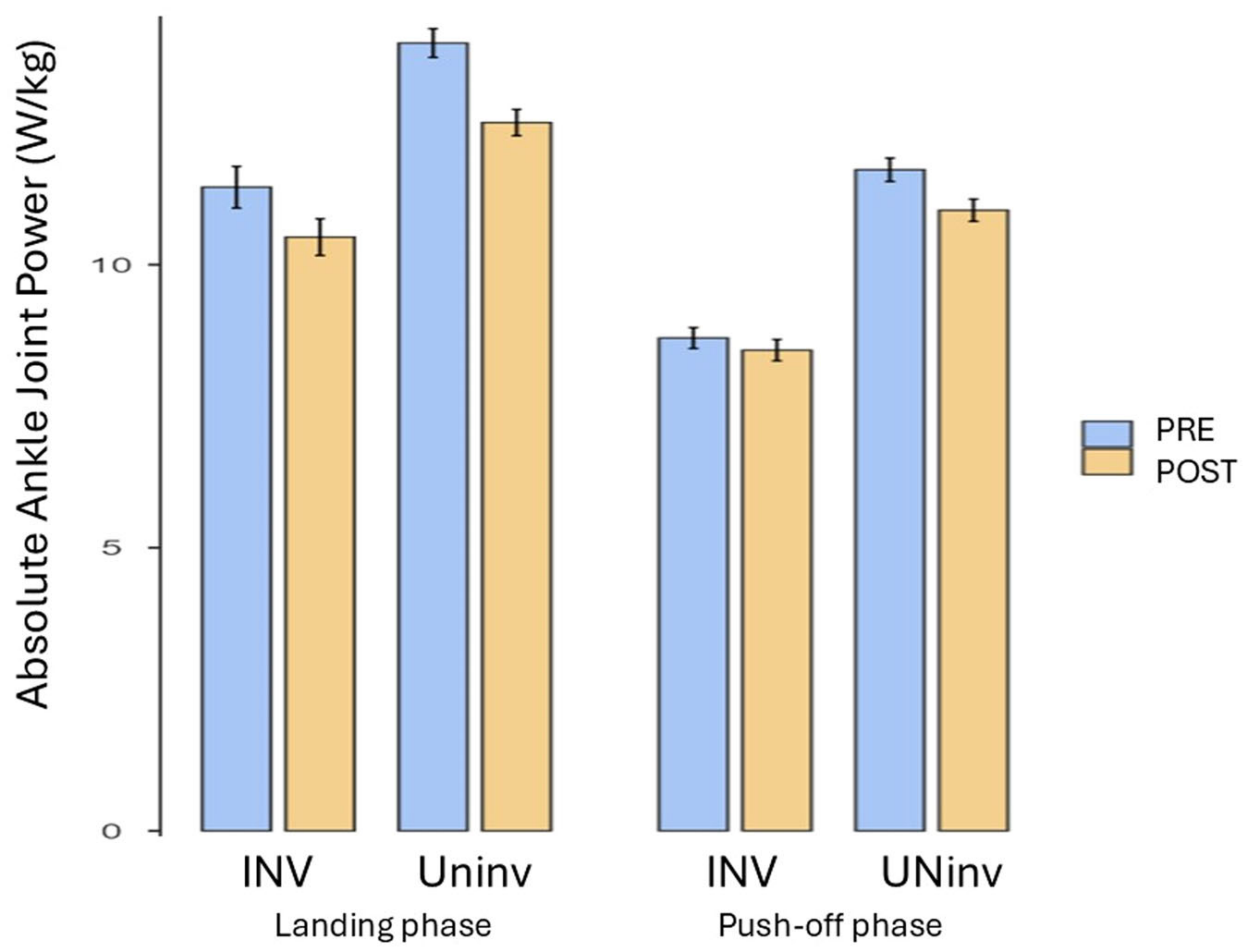
Pre‐ to post‐fatigue change in ankle joint power (mean W/kg with 95% CI) during single‐limb drop CMJ, reflecting a fatigue by side interaction (greater post‐fatigue drop of the uninvolved than the involved side across both phases), and a fatigue by phase interaction (greater post‐fatigue drop during landing compared push‐off across both sides). CI, confidence interval; CMJ, countermovement jump; INV, involved side; UNinv, uninvolved side.

At the knee joint, main effects show the INV side to produce overall greater knee joint power than the UNinv (*p* < 0.001) and generally greater eccentric (landing) versus concentric (push‐off) power (*p* < 0.001). Only a trend towards increased knee joint power was seen as a response to fatigue across both sides and phases (*p* = 0.062). At the hip, the only statistical finding in either plane was a main effect of phase, showing greater power during landing compared to push‐off (*p* < 0.001), regardless of side or fatigue.

## DISCUSSION

The most important results of the study demonstrated that the fatigue protocol significantly affected ankle joint power, but primarily for the UNinv side. Effects of fatigue on physical performance were further reflected by a more marked reduction in jump height and speed of performance of the UNinv compared to the INV side. Overall, a proximal shift of joint power from the ankle to the knee was seen on the INV side, regardless of fatigue or CMJ phase.

Deficits in single‐leg jump height after ATR, regardless of surgical versus non‐surgical management, have been previously reported in the literature [24, 27]. The reasons for long‐term effects of ATR on performance of the INV side (jump height, peak ankle joint power and time‐to‐peak) have been attributed to structural changes such as tendon elongation and loss of the normal anatomical rotation prior to insertion onto the calcaneus [11, 22, 34] and altered muscle activation patterns [33]. Such structural changes will likely negatively affect the tendon's energy storage and return during dynamic activities and may have prompted the proximal joint compensation seen at the INV knee. This is further supported by the heel‐rise test performance numbers, where deficits in heel‐rise height were more prominent than the number of completed repetitions. The distal‐to‐proximal shift and the greater effect of ATR on the landing vs. push‐off phase during jumping have previously been shown [28] and should be kept in mind during rehabilitation in terms of lower limb conditioning and task‐specific training, and in cases where overuse injuries might emerge. The reduction of ankle joint power on the UNinv side was unexpected and may reflect overreliance of that side during tasks that involve both lower extremities and/or inhibition of the INV side, leading to selective fatigue on the UNinv side during the slide board task. Rehabilitation after ATR may consider providing feedback that helps promote bilateral performance during double‐stance tasks. This would promote reliance and confidence in the use of the INV side plantar flexors and reduce compensation on the UNinv side, which might have further benefits of reducing the risk of kinesiophobia, which has been shown to negatively influence performance [20]. Most rehabilitation protocols (regardless of medical management) focus on the earlier phases in terms of starting weight‐bearing and range of motion and are known to employ a large variety of interventions and outcome measures [36]. When initiating weight‐bearing, heel wedges are used to reduce loading at the healing tendon, but the concomitant effect of each added wedge is to incrementally shift loading to the knee extensors [37]. This should be remembered when transitioning to mid‐ and late‐stage rehabilitation, where the use of biomechanically minded exercises can strategically reduce compensatory, proximal shifts of joint work during closed‐chain lower‐limb training to optimize recovery at the ankle. Another consideration includes muscle activation levels after ATR, where a greater contribution from the soleus than the gastrocnemius has been identified during single‐leg hopping using electromyography (EMG) [15].

As noted in the introduction, the effects of fatigue are relatively unknown in this population. The fact that the fatigue protocol had a greater effect on the UNinv side's ankle joint power was somewhat unexpected and may reflect greater reliance and exertion of participants on that side, although limb‐specific exertion and fatigue were not measured to confirm that. Considering the literature, one might have expected a greater proximal shift of joint power from the ankles to the knees and hips [10], and there was a trend seen towards greater knee joint power post‐fatigue, bilaterally, although the change did not reach statistical significance. Variability in fatigue protocols and testing procedures likely influences such outcomes [3]. Between the pre‐ and post‐fatigue biomechanical measurements, the participants underwent two forms of fatigue interventions. The heel‐rise test targets the plantar flexors specifically, but one of the criteria to stop the test uses heel‐rise height. Deficits of 16%–17% in heel‐rise height after ATR are known to persist [26], and this may impact the magnitude of muscle exhaustion during the heel‐rise test on the INV side. During lateral slides, some involuntary hesitancy or inhibition of performance on the INV side may also have led to greater exertion on the UNinv side. No specific measurements were done to evaluate limb‐specific perception of exertion after each test. Clinical implications of the results concerning effects of fatigue include the consideration of implementing training under fatigued states during late‐stage rehabilitation. This might enable clinicians to better identify potential compensatory strategies and to design targeted, personalized training programs that promote confidence in the INV side and encourage effective movement strategies, even after fatigue kicks in.

Limitations to the study include those of the fatigue protocol, as noted earlier, although the performance measures and self‐reported scale indicate that a level of fatigue was reached. The fixed test order of the drop CMJ and heel‐rise test could have introduced an effect between limbs. The INV side goes into the exertion task on the slide board task, having just completed heel‐rises to exhaustion, which may have influenced bilateral performance. On the other hand, the UNinv side goes from the slide board straight to the second drop CMJ task, which may have contributed to the inter‐limb differences seen, although the delay between testing the UNinv and INV was very short. The main outcome measure was captured during a task that is not very sports‐like (single‐limb drop CMJ) but was chosen to identify changes in joint power. To truly appreciate changes in muscle and lower limb function, EMG measures would have been necessary. A larger cohort would have provided greater statistical power for subgroup analysis based on, for example, time since injury/surgery and surgical vs. non‐surgical management, but this was not the primary focus of the study. The use of tendon length symmetry as a covariate was used to at least capture some structural effects related to management. The nature of participants' rehabilitation after injury/surgery was unknown, and another limitation includes varied levels of cardiovascular fitness, which may influence their rating of fatigue [5].

## CONCLUSION

The results indicate that the local and general fatigue protocol induced changes primarily on the UNinv side, potentially due to compensatory over‐reliance on that side. This may be addressed strategically during all phases of rehabilitation by considering principles of biomechanics and utilizing biofeedback to improve confidence and reliance on the INV side for optimal recovery. Further research is required to fully capture effects of fatigue on lower limb performance and muscle activation after ATR and the impact of specific rehabilitation strategies.

## AUTHOR CONTRIBUTIONS

Roy Tranberg, Karin Grävare Silbernagel, Annelie Brorsson and Kristín Briem designed the study. Roy Tranberg, Annelie Brorsson and Kristín Briem conducted all data collections. Unnur Sædís Jónsdóttir, Roy Tranberg and Kristín Briem processed the data. Unnur Sædís Jónsdóttir and KB did statistical analysis. All authors contributed to decisions regarding data presentation and writing of the manuscript.

## CONFLICT OF INTEREST STATEMENT

The authors declare no conflicts of interest.

## ETHICS STATEMENT

This study was approved by the Regional Ethical Review Board in Gothenburg, Sweden (nr 803‐15). Prior to enrolment and data collection, information about the research was provided, and participants gave oral and written consent.

## Data Availability

The data that support the findings of this study are available from the corresponding author upon reasonable request.
